# Impact of yak excreta on soil bacterial community in alpine marsh under warming conditions

**DOI:** 10.1128/aem.01493-25

**Published:** 2025-10-21

**Authors:** Xuelian Guo, Chen Yang, Qian Fu, Hanbing Li, Jian Fang, Rongbo Zheng

**Affiliations:** 1 School of Environment and Natural Resources, Zhejiang University of Science and Technology 91616, Hangzhou, China; 2 Zhejiang Province Key Laboratory of Recycling and Ecological Treatment of Waste Biomass, Hangzhou, China; 3 School of Biological and Chemical Engineering, Zhejiang University of Science and Technology 91616, Hangzhou, China; University of Delaware, Lewes, Delaware, USA

**Keywords:** yak excreta, bacterial community, marsh soil, climate warming

## Abstract

Livestock excreta and climate warming are two main disturbances of wetlands embedded in grazing lands, resulting in long-lasting changes in soil microorganisms. However, the impact of livestock excreta on the soil bacteria community in wetlands with climate warming has not been elucidated. In the current study, a laboratory culture experiment was designed to investigate how yak excreta, temperature, and their interaction regulate the soil bacterial community in an alpine marsh. The results show that yak dung increased soil moisture, pH, total organic carbon (TOC), and available phosphorus (AP), but decreased NO_3_^−^–N (*P* < 0.05). Yak urine increased soil moisture, NH_4_^+^–N, and NO_3_^−^–N (*P* < 0.05). Warming decreased soil moisture and pH of marsh soil (*P* < 0.05). Warming increased the alpha-diversity of the bacterial community in marsh soil; yak dung had an opposite effect, while yak urine exerted almost a negligible effect. In comparison with warming, yak excreta was the main cause of changing the bacterial community in marsh soil. Yak dung altered more bacterial genera of marsh soil than yak urine. Moreover, yak dung obviously strengthened the bacterial association interaction in marsh soil, while yak urine had the opposite trend. Yak excreta and temperature altered the bacterial community by regulating NO_3_^−^–N, AP, pH, TOC, and moisture of marsh soil. This study confirms the different influences of yak dung and urine on the bacterial community of marsh soil under warming conditions and highlights that the impacts of yak excreta on the bacterial community are sensitive to climate warming.

**IMPORTANCE:**

Investigating the response of the bacterial community in marsh soil to external disturbances is an important but poorly elucidated topic in microbial ecology. In this study, we evaluated the impacts of yak excreta, temperature, and their interaction on the bacterial community in alpine marsh soil. Our results showed that yak excreta exhibited a stronger influence on the bacterial community of marsh soil than temperature. The response of the bacterial community of marsh soil to yak dung is more sensitive than to yak urine. Yak excreta and temperature significantly altered the bacterial community by regulating NO_3_^−^–N, AP, pH, TOC, and moisture of marsh soil. Understanding the impact of yak excreta on soil bacterial community under warming conditions is extremely significant for managing grazing and maintaining a healthy alpine marsh ecosystem.

## INTRODUCTION

Wetlands play important roles in maintaining biological diversity and regulating the climate (1). However, it has been degraded significantly due to both climatic and human activities over the past century (2, 3). Livestock grazing is one of the main disturbances of wetlands embedded in grazing lands, resulting in long-lasting changes in plants, soil, and microorganisms (4). Although the impact of livestock grazing on wetlands has been studied in depth and synthesized (4–6), the research on livestock excreta impacts on wetlands is more limited (7–9). This is particularly the case when livestock excreta is combined with climate warming, which may interact with livestock excreta to affect wetlands. Therefore, to understand the impact of livestock excreta on wetlands with climate warming is essential for wetland conservation.

Bacterial community is an important component of wetland ecosystems and plays a crucial role in maintaining the function and stability of wetland ecosystems, including decomposing organic matter, promoting biogeochemical cycling of elements, and regulating plant nutrient availability (3, 10). Bacterial community is very sensitive to environmental change, such as temperature fluctuation, water content change, nutrient input, and other changes in soil properties (11–13). Warming directly impacts soil bacterial activities and thus soil carbon sequestration, and thereby affects future climate change (14, 15). In addition, warming also strongly alters soil bacterial communities by altering soil pH, soil nutrient availability, and plant community composition and productivity (16, 17). Livestock excreta directly inputs microorganisms into it (18) and indirectly alters the physical, chemical, and biological characteristics of the soil after its deposition, thus altering the composition and diversity of the soil bacterial community (19, 20). Livestock dung is rich in water, organic matter, and microbes, and their deposition in soil has a more complex effect on bacterial communities than urine (21). However, the key differences between the effects of livestock dung and urine on bacterial communities in wetland ecosystems under warming conditions have not been elucidated.

Grazing is one of the main disturbances in marshes in the northwest Yunnan plateau, situated at the southeastern rim of the Qinhai-Tibetan Plateau of China (8). Yak is the dominant livestock species in the northwest Yunnan Plateau. It is growing rapidly and is now significantly higher than estimated (22). In the context of global warming, the temperature in the northwest Yunnan Plateau has risen by more than 2°C over the past 50 years, and the effects of warming on high-elevation marshes may be complex and poorly understood (23). Therefore, it is necessary to determine how bacterial communities in the marsh soils of the northwest Yunnan Plateau respond to yak excreta addition in the context of global warming.

The specific objective of the study was to assess the influence of yak excreta addition and temperature changes on the bacterial community structure of mash soil. We hypothesize that (i) yak excreta exhibited a stronger influence on the bacterial community in marsh soil than temperature; (ii) yak dung will exert stronger effects than yak urine due to higher nutrient and microbial load, and that warming will modulate these effects by altering soil moisture and nutrient turnover. These study results could provide novel insights into the different impacts of yak dung and urine on the bacterial community of marsh soil under warming conditions and highlight the distinct responses of bacterial communities in marsh soil and marsh soil with yak excreta to climate warming.

## MATERIALS AND METHODS

### Soil and yak excreta sampling

Samples of soil were gathered from the grazing marsh located in the Napahai wetland in Southwest China (27°47′–27°55′N，99°35′–99°43′E; elevation 3,260 m), in the southeast Qinghai-Tibetan Plateau. The site is located in the intersection of the Tibetan Plateau, subtropical monsoon, and the Indo-China Peninsula monsoon climate zones. The annual average temperature is 5.4°C, the mean, peak, and extremely high summer temperatures are 13°C, 19°C, and 25°C. The annual average rainfall stands at roughly 619.9 mm, with about 76% occurring between June and September.

Three duplicated plots (10 m × 20 m) were set up and spaced 10 m apart. Post-litter removal, soil samples were gathered from 0 to 10 cm depths of each plot using a hand auger (2.5 cm in diameter) in an S-shaped formation. For acquiring representative samples, 10 samples from each plot were collected and combined to create a unified composite sample, which was then promptly stored in plastic bags and transported to the laboratory. Ten yaks located on a camping site near the research area were selected. At night, the camp housed yaks, and the following morning, fresh dung and urine samples were gathered manually into plastic containers. All fecal matter gathered from yaks was meticulously blended and stored in a fridge at 4°C before being applied to the soil. The chemical characteristics of the soil and yak excreta samples are determined and shown in Table S1 at https://doi.org/10.5281/zenodo.17188844.

### Incubation experiment

The composite soil was air-dried, homogenized, and sieved through a 2 mm mesh. Microcosms were prepared with 100 g of sieved soil in a 500 mL Erlenmeyer flask. Every incubation flask was sealed with a butyl rubber stopper, positioned within the incubators, and subsequently pre-incubated at 25°C for a week in darkness. In this study, the trio of experimental groups included marsh soil (CK), a combination of yak dung and marsh soil (DS), and a combination of yak urine and marsh soil (US). The quantity of dung added was determined through field investigation. The addition of urine followed the mean volume of urine documented by van Groenigen (24) during field grazing. A consistent application of urine (2.35 mL, equivalent to 56.28 mL·kg^−1^) and dung (17.25 g, equivalent to 419 g·kg^−1^) was applied to the marsh soil’s surface. Every incubation flask, sealed with a rubber plug, was positioned in culture containers following a randomized block pattern at temperatures of 13°C, 19°C, and 25°C (the mean, peak, and extremely high temperatures in the summer of the study site). The flasks were unsealed to enable the headspace to balance with lab air, ensuring daily aerobic conditions. Soil samples were periodically harvested through destructive sampling after incubation periods of 1 and 28 days, and the physicochemical characteristics and bacterial community of them were measured.

### Measurement of soil physicochemical properties

The moisture of soil samples was evaluated by drying method at 105°C. Soil pH was measured with a pH meter (Ohaus STARTER3100, USA). Total organic carbon (TOC) content was analyzed by using a total organic carbon analyzer (Elementar, Germany). Total nitrogen (TN) and total phosphorus (TP) were analyzed by a continuous flow analyzer (Seal AA3, Norderstedt, Germany). NH_4_^+^–N and NO_3_^−^–N were extracted from fresh soil using a 1 mol/L KCl mixture (with a soil to solution ratio of 1:5) and subsequently analyzed using a continuous flow analyzer (Seal AA3, Norderstedt, Germany). The molybdate blue colorimetric technique was employed to analyze soil available phosphorus (AP).

### DNA extractions and Illumina MiSeq sequencing

Microbial DNA was isolated from 0.5 g of fresh soil samples by a Soil DNA Kit (Omega Bio-Tek, Norcross, GA, USA). The ultimate concentration and refinement of DNA were measured using a NanoDrop 2000 UV-vis spectrophotometer (Thermo Scientific, Wilmington, USA), with DNA integrity verified through 1% agarose gel electrophoresis. Using primers 338F (5′-ACTCCTACGGGAGGCAGCAG-3′) and 806R (5′-GGACTACHVGGGTWTCTAAT-3′) from the GeneAmp 9700 thermocycler PCR system (ABI, USA), the V3–V4 highly variable segments of the bacterial 16S rRNA gene were magnified. The PCR products were extracted from a 2% agarose gel, purified with the AxyPrep DNA Gel Extraction Kit (Axygen Biosciences, Union City, CA, USA), and measured using QuantiFluor-ST (Promega, USA), adhering to Illumina MiSeq sequencing guidelines.

Purified amplicons were combined using equimolar and paired-end sequencing (2 × 300) on an Illumina MiSeq system (Illumina, San Diego, USA), adhering to the established procedures of Majorbio Bio-Pharm Technology Co. Limited (Shanghai, China). Utilizing UPARSE (version 7.1 http://drive5.com/uparse/), operational taxonomic units (OTUs) underwent clustering with a 97% similarity threshold, employing an innovative “greedy” algorithm for concurrent chimera filtering and OTU clustering. The 16S rRNA gene sequences were processed and taxonomically classified using the SILVAngs analysis platform (https://www.arb-silva.de/ngs/), following the platform’s standard pipeline, which includes quality control and alignment against the SILVA SSU rRNA reference dataset.

### Statistical analysis

One-way analysis of variance (ANOVA) (*P* < 0.05) along with Tukey’s test was used to identify notable variances among soil physicochemical properties and bacterial community diversity, while a two-way ANOVA analyzed the impact of excreta addition, temperature, and interaction on soil physicochemical properties and bacterial community diversity among different treatments. The association between soil physicochemical properties and bacterial community diversity was assessed using Pearson correlation coefficient analysis. The connection between bacterial community and environmental elements was discerned using Spearman and Canonical correlation analyses (CCA). SPSS 27.0 (IBM Software, Chicago, IL, USA) was utilized to conduct ANOVA and Tukey’s test. The CCA procedure was executed utilizing Canoco 4.5 (Microcomputer Power Inc., Ithaca, NY, USA). Visualization of the experimental data was achieved through the use of OriginPro 2021 (OriginLab Inc., USA) for both graphing and plotting purposes.

## RESULTS

### Soil physical and chemical properties

Moisture showed a decreasing trend at 13°C and 25°C: DS > US > CK (*P* < 0.05) (Table 1). pH of DS was higher than US and CK (*P* < 0.05). TOC and AP of DS were higher than US and CK (*P* < 0.05). TN of DS was higher than US and CK at 13°C (*P* < 0.05). NO_3_^−^–N showed a decreasing trend: US > CK > DS (*P* < 0.05). NH_4_^+^–N of the US was significantly higher than DS and CK (*P* < 0.05).

**TABLE 1:** Physicochemical properties of marsh soil with yak excreta addition under warming condition^*a*^

Treatment		Moisture (%)	pH	TOC (g·kg^−1^)	TN (g·kg^−1^)	TP (g·kg^−1^)	NH_4_^+^–N (mg·kg^−1^)	NO_3_^−^–N (mg·kg^−1^)	AP (mg·kg^−1^)
13°C	CK	39.58 ± 0.16 Ac	5.70 ± 0.14 Ab	94.98 ± 3.83 Ab	7.36 ± 0.41 Aab	6.60 ± 0.21 Aa	45.18 ± 9.29 Bb	212.39 (16.37) Ab	4.99 ± 0.13 Ab
	DS	60.38 ± 0.14 Aa	6.55 ± 0.05 Aa	112.68 ± 1.50 Aa	7.88 ± 0.31 Aa	6.46 ± 0.14 Ba	117.15 ± 53.90 Ab	34.16 (1.90) Ac	22.64 ± 0.77 Aa
	US	44.68 ± 0.05 Ab	5.99 ± 0.16 Ab	91.87 ± 2.20 Ab	6.47 ± 0.29 Ab	6.52 ± 0.18 Ba	1302.10 ± 11.34 Aa	254.02 (9.28) Ca	3.53 ± 0.29 Bb
19°C	CK	38.61 ± 0.27 Ba	5.22 ± 0.01 Bb	97.47 ± 2.98 Ab	7.68 ± 1.74 Aa	6.58 ± 0.22 Aa	17.52 ± 1.48 Bb	245.88 (3.97) Ab	5.26 ± 0.91 Ab
	DS	53.00 ± 7.28 Aa	6.28 ± 0.03 Ba	117.09 ± 4.02 Aa	6.98 ± 0.83 Aa	7.20 ± 0.07 Aa	19.86 ± 0.71 Bb	21.96 (0.61) Bc	23.66 ± 3.29 Aa
	US	47.20 ± 2.93 Aa	4.87 ± 0.04 Cc	94.90 ± 2.01 Ab	7.10 ± 0.15 Aa	6.64 ± 0.22 ABa	336.51 ± 16.02 Ca	313.40 (2.03) Aa	5.12 ± 1.16 ABb
25°C	CK	37.66 ± 0.30 Cc	5.34 ± 0.01 Bb	90.32 ± 1.41 Ab	6.61 ± 0.70 Aa	7.08 ± 0.12 Aa	120.97 ± 14.46 Ab	211.41 (14.55) Ab	4.55 ± 0.44 Bb
	DS	61.82 ± 1.74 Aa	6.44 ± 0.06 Aa	117.52 ± 1.44 Aa	7.59 ± 0.82 Aa	6.63 ± 0.31 ABa	43.78 ± 8.53 Bb	30.49 (1.66) Ac	20.96 ± 2.53 Aa
	US	43.48 ± 0.10 Ab	5.31 ± 0.13 Bb	88.80 ± 0.71 Ab	6.75 ± 0.35 Aa	7.15 ± 0.07 Aa	704.66 ± 111.90 Ba	290.84 (2.32) Ba	8.25 ± 1.35 Ab

^*a*^ CK, marsh soil; DS, yak dung + marsh soil; US, yak urine + marsh soil. Lowercase letters indicate significant differences (*P *< 0.05) among treatments of yak excreta addition, while uppercase letters represent significant differences (*P *< 0.05) among treatments of temperature.

Moisture of CK exhibited a decreasing trend: 13°C > 19°C > 25°C (*P* < 0.05). pH of CK was higher at 13°C than 19°C and 25°C (*P* < 0.05). pH of DS was lower at 19°C than at 13°C and 25°C (*P* < 0.05), but that of US showed a decrease: 13°C > 25°C > 19°C (*P* < 0.05). NH_4_^+^–N of CK was higher at 25°C than 13°C and 19°C, and that of US exhibited the following trend: 13°C > 25°C > 19°C (*P* < 0.05). In contrast, NO_3_^−^–N of the US exhibited the following trend: 19°C > 25°C > 13°C (*P* < 0.05). AP of CK was significantly higher at 13°C and 19°C than at 25°C (*P* < 0.05). AP of US was higher at 25°C than at 13°C and 19°C (*P* < 0.05). TP of DS was lower at 13°C than 19°C (*P* < 0.05), and that of US was lower at 13°C than 25°C (*P* < 0.05).

Yak excreta significantly altered moisture, pH, TOC, TN, NH_4_^+^–N, and NO_3_^−^–N (*P* < 0.01), both temperature and yak excreta and temperature interactions greatly changed pH, NH_4_^+^–N, and NO_3_^−^–N (*P* < 0.01) (see Table S2 at https://doi.org/10.5281/zenodo.17188844).

### Soil bacterial community diversity

**Fig 1:**
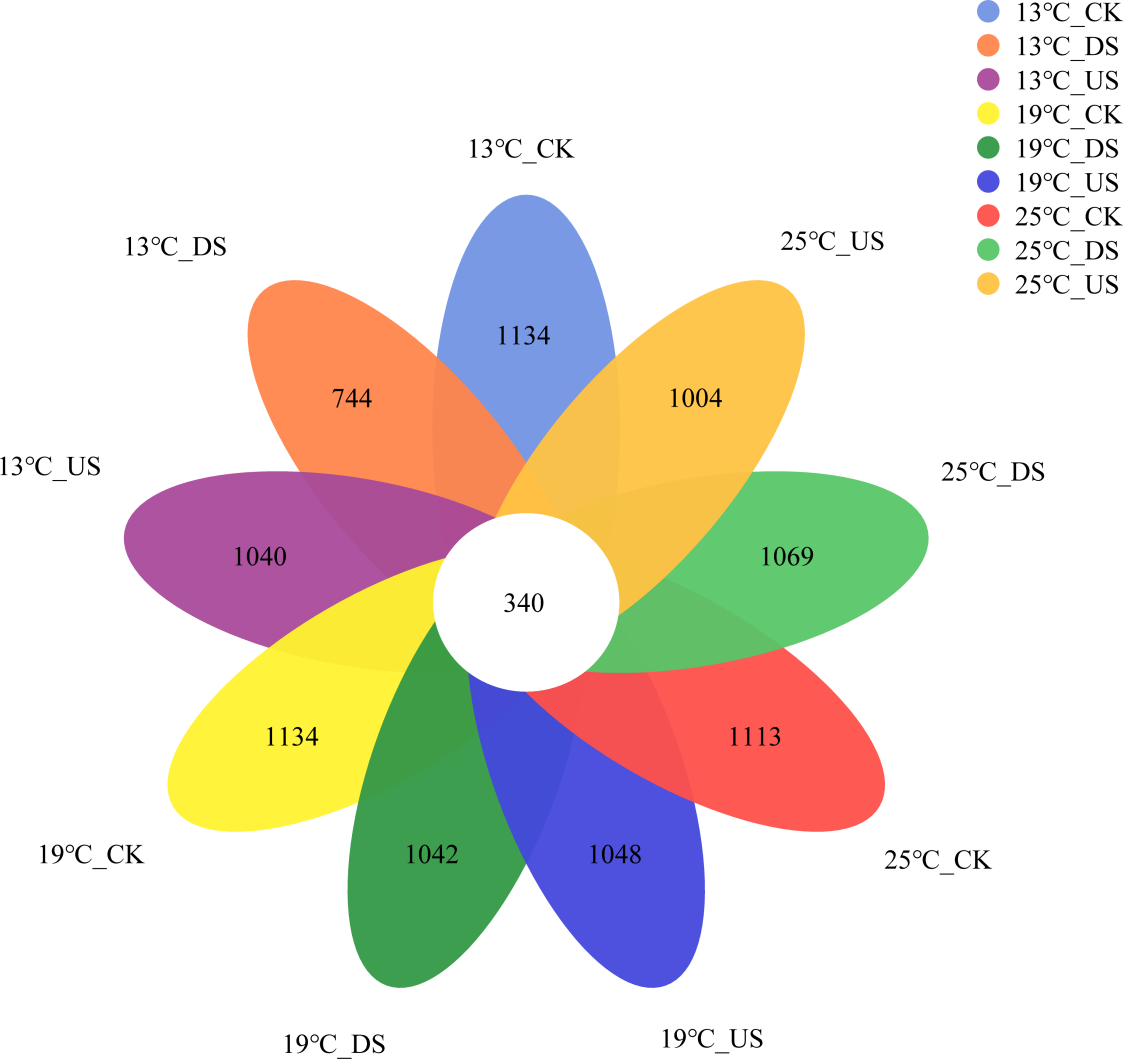
Venn diagram analysis of bacterial communities in marsh soil with yak excreta addition (CK, marsh soil; DS, yak dung + marsh soil; US, yak urine + marsh soil) under warming conditions. The number in each petal represents the number of unique OTUs in the corresponding group, and the number in the center represents the number of common OTUs in all groups.

**Fig 2:**
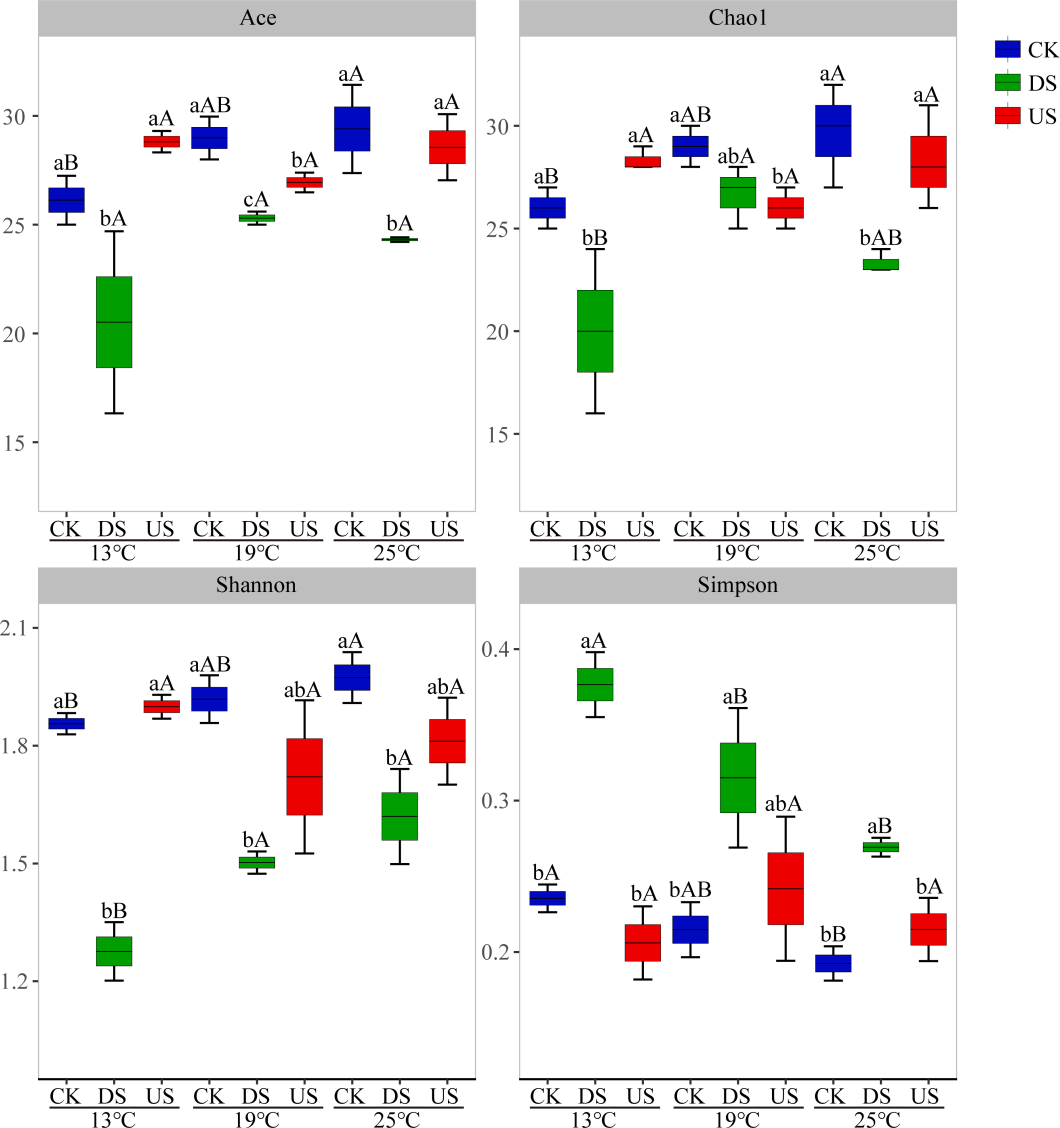
The alpha-diversity indices of bacterial communities in marsh soil with yak excreta addition (CK, marsh soil; DS, yak dung + marsh soil; US, yak urine + marsh soil) under warming conditions. Lowercase letters indicate significant differences (*P* < 0.05) among treatments of yak excreta addition, while capital letters represent significant differences (*P* < 0.05) among treatments of temperature.

The number of common OTUs among the different treatments was 340 (Fig. 1). The unique OTU numbers of CK at different temperatures were higher than DS and US. The unique OTUs of DS increased with the temperature rise. Alpha diversity analysis revealed that yak excreta and temperature both had a significant impact on bacterial diversity in marsh soil (Fig. 2). Ace, Chao1, and Shannon index of DS were lower than those of CK. In contrast, the Simpson index of DS was higher than that of CK (*P* < 0.05). Ace and Chao1 indices of the US were lower than those of CK at 19°C (*P* < 0.05). Ace, Chao1, and Shannon indices of CK were higher at 25°C than at 13°C, while the Simpson index of CK was lower at 25°C than at 13°C (*P* < 0.05). Shannon index of DS was lower at 13°C than at 19°C and 25°C, whereas Simpson index exhibited a reverse pattern (*P* < 0.05). The bacterial diversity index was mainly affected by yak excreta (*P* < 0.01, Table S2 at https://doi.org/10.5281/zenodo.17188844).

### Soil bacterial community structure and co-occurrence network

**Fig 3:**
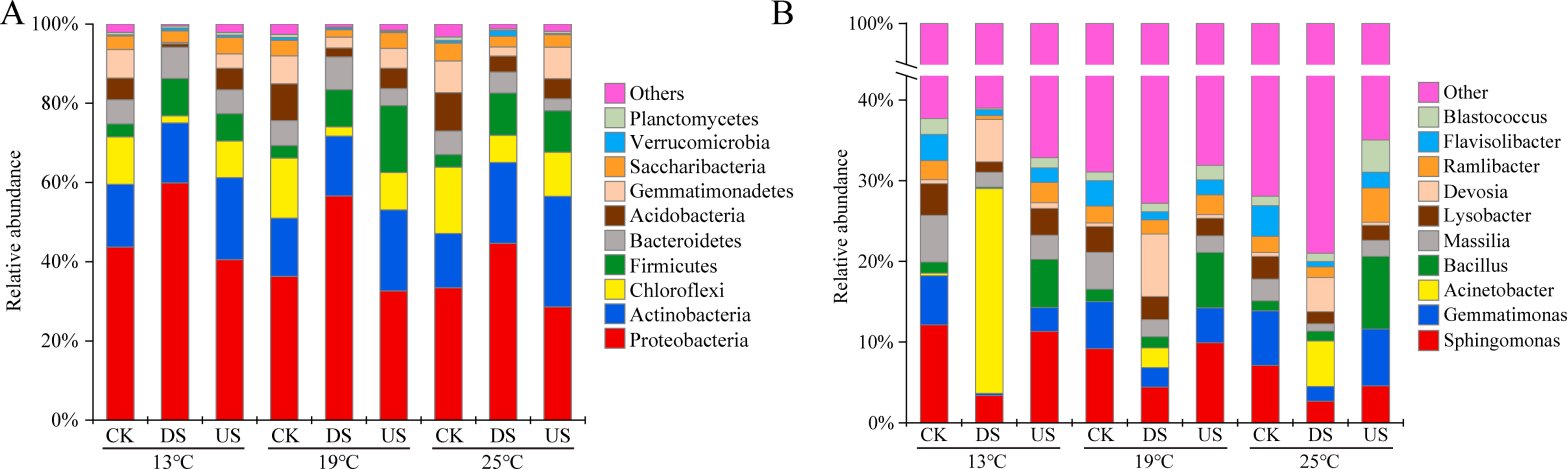
The relative abundance of dominant (top 10) bacterial phylum (**A**) and genus (**B**) in marsh soils with yak excreta addition (CK, marsh soil; DS, yak dung + marsh soil; US, yak urine + marsh soil) under warming conditions.

Within the phylum classification, *Proteobacteria*, *Actinobacteriota,* and *Chloroflexi* were predominant phyla in the marsh soil under varied treatments, which accounted for over 60% (Fig. 3A). DS increased the proportion of *Proteobacteria*, but US resulted in the reverse effect. Yak excreta decreased the proportion of *Chloroflexi*, *Acidobacteria*, and *Gemmatimonadetes,* while increasing the proportion of *Firmicutes* (Fig. 3A, also see Fig. S1 at https://doi.org/10.5281/zenodo.17188844). As the temperature rose, the percentage of *Proteobacteria* decreased across various treatments. The percentage of *Chloroflexi*, *Verrucomicrobia*, and *Planctomycetes* increased when the temperature increased from 13°C to 25°C (*P* < 0.01, Fig. S1 at https://doi.org/10.5281/zenodo.17188844).

In terms of the genus level, DS decreased the percentage of *Sphingomonas*, *Gemmatimonas*, *Massilia*, *Lysobacter*, and *Flavisolibacter* while increasing the percentage of *Acinetobacter* and *Devosia*. In contrast, the US increased the percentage of *Bacillus*. The proportion of *Acinetobacter* in DS showed a decreasing trend: 13°C > 25°C > 19°C. As compared to CK, DS significantly altered 16 bacterial genera, and US changed five bacterial genera. There are three bacterial genera that changed when the temperature increased from 13°C to 19°C, and eight genera changed when the temperature increased from 13°C to 25°C (see Fig. S1 at https://doi.org/10.5281/zenodo.17188844). The relative abundances of dominant bacterial phyla and genera were mainly affected by yak excreta and yak excreta and temperature interactions (*P* < 0.05, see Table S3 at https://doi.org/10.5281/zenodo.17188844).

**Fig 4:**
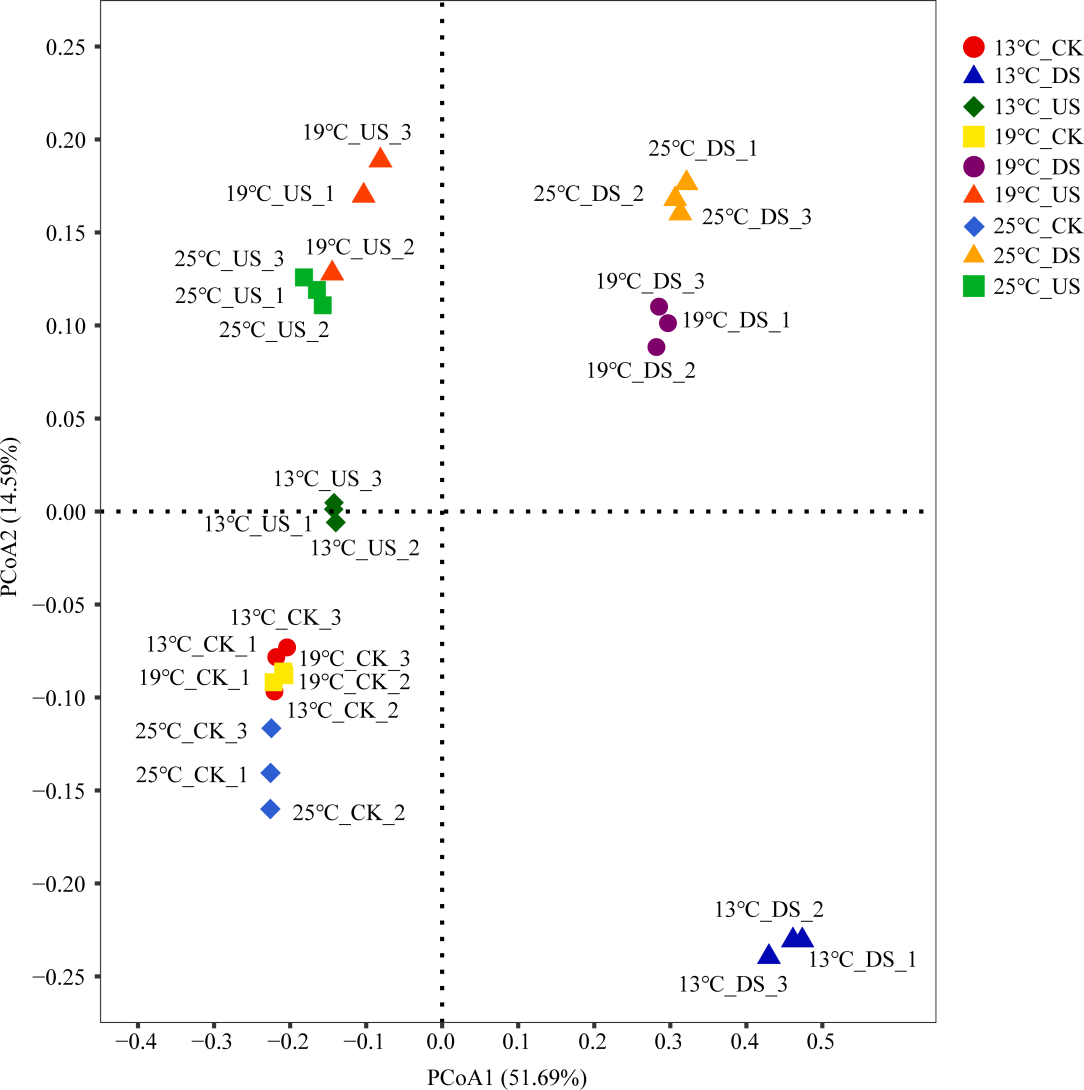
PCoA of bacterial 16S rRNA gene sequencing in marsh soil with yak excreta addition (CK, marsh soil; DS, yak dung + marsh soil; US, yak urine + marsh soil) under warming conditions.

The principal coordinates analysis (PCoA) revealed that the PCoA1 and PCoA2 axes interpreted the results at rates of 51.69% and 14.59%, respectively (Fig. 4). Findings indicated that CK samples were categorized on the figure’ left, DS samples were grouped on the right side of the figure by the first principal component (PCoA1), and US samples on the top by the second principal component (PCoA2). US and DS samples at 13°C were separated from those at 19°C and 25°C.

**Fig 5:**
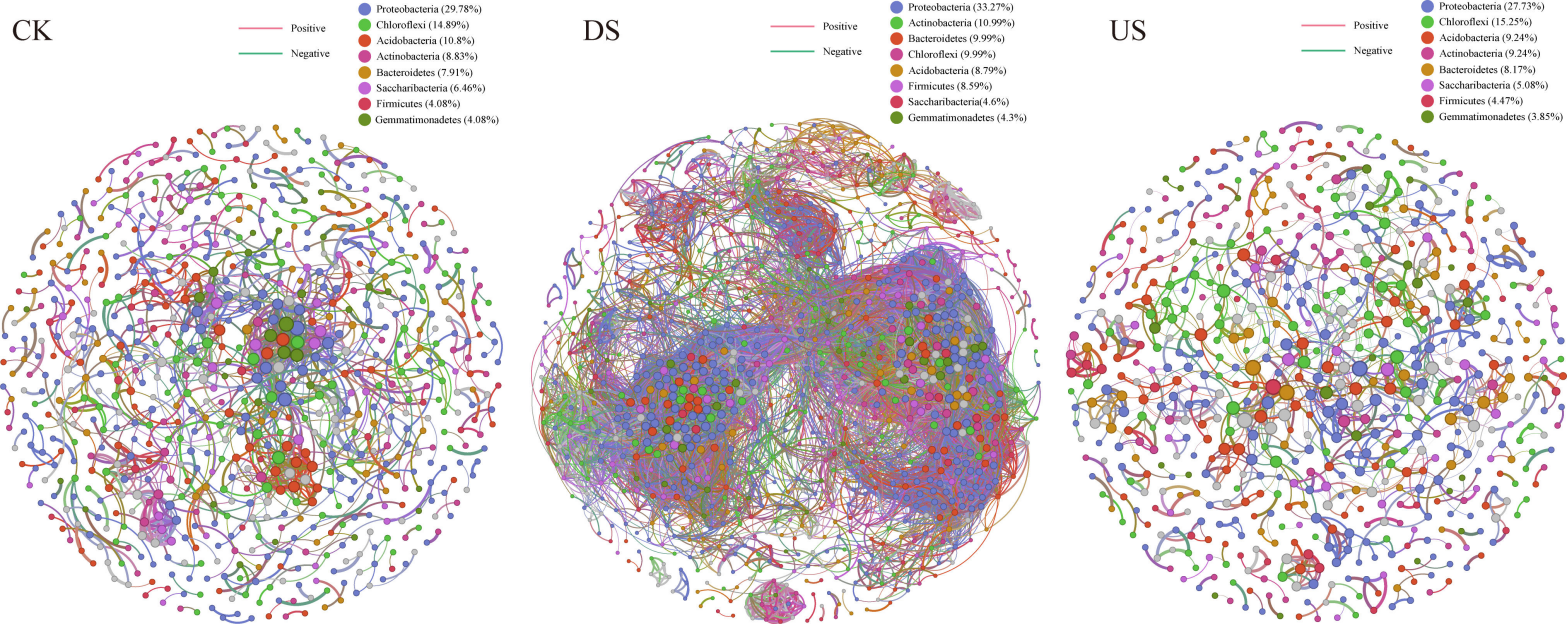
The co-occurrence networks of soil bacterial community at the phylum level in marsh soils with yak excreta addition (CK, marsh soil; DS, yak dung + marsh soil; US, yak urine + marsh soil).

Co-occurrence networks were constructed to examine whether there were differences in topographic features of bacterial community at the phylum level in marsh soil with yak excreta treatments (Fig. 5; Table 2). The highest number of bacterial network nodes (1,001) and edges (19,105) were found in DS, while the lowest number of bacterial network nodes (649) and edges (1,084) were found in US. However, we found more positive interactions among bacterial networks in DS (83.63%) and US (97.05%) compared to CK (77.12%). In comparison, lower negative interactions among bacterial networks in DS (16.37%) and US (2.95%) compared to CK (22.88%) were found (Table 2). *Proteobacteria* was the predominant phylum identified in the bacterial networks across different yak excreta treatments. The fewest proportion of *Chloroflexi* and the highest proportion of *Firmicutes* were found in DS (Fig. 5).

**TABLE 2:** Network properties of soil bacterial community at phylum level in marsh soils with yak excreta addition^*a*^

Properties	Nodes	Edges	Average degree	Network diameter	Average path length	Graph density	Modularity	Clustering coefficient	Positive interactions (%)	Negative interactions (%)
CK	759	1,259	3.318	26	6.766	0.004	0.858	0.472	77.12	22.88
DS	1,001	19,105	38.172	15	4.993	0.038	0.617	0.648	83.63	16.37
US	649	1,084	3.341	30	9.191	0.005	0.895	0.588	97.05	2.95

^*a*^ CK, marsh soil; DS, yak dung + marsh soil; US, yak urine + marsh soil.

### Correlation between soil properties and bacterial community diversity

**Fig 6:**
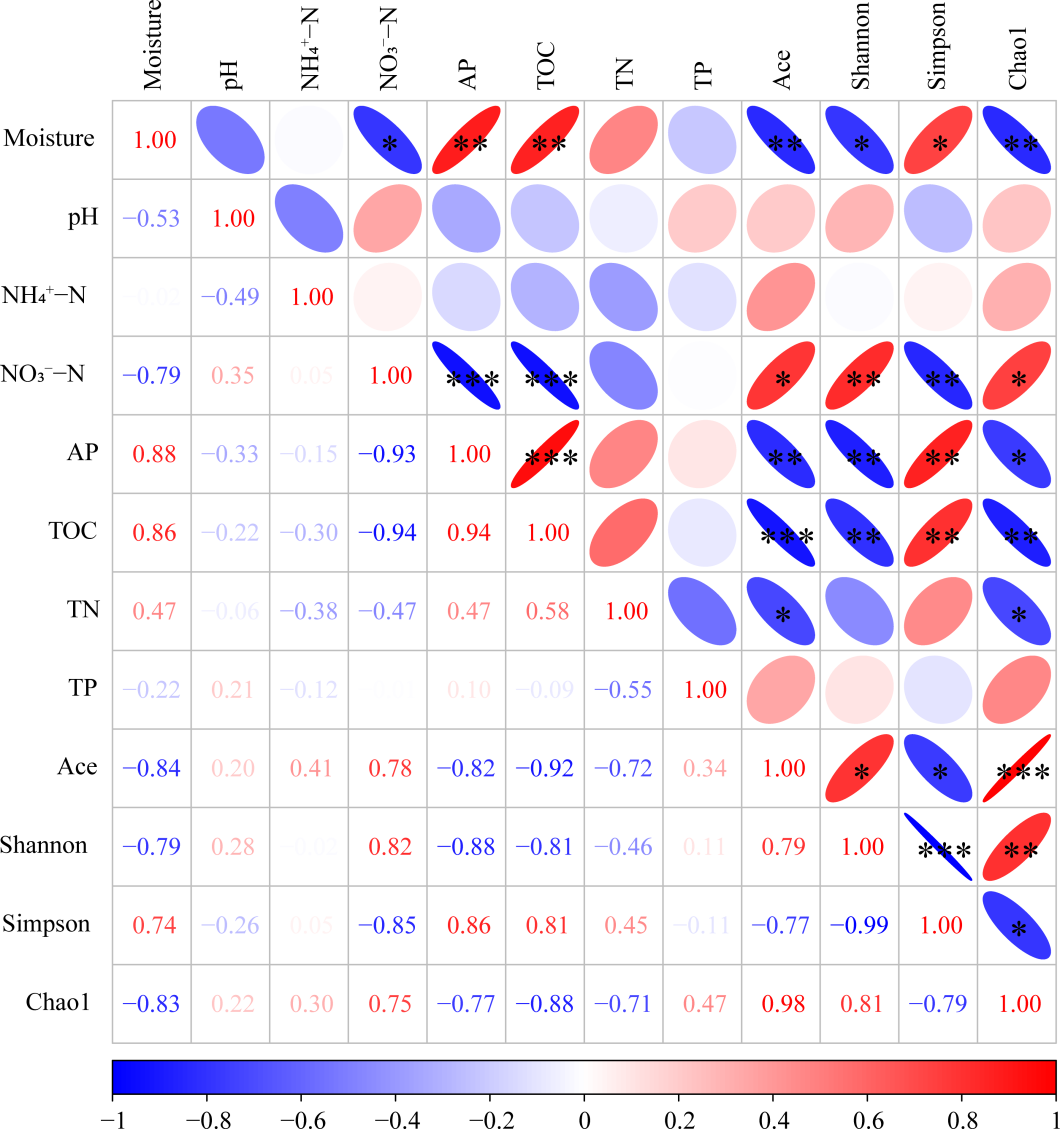
Correlation matrix showing Pearson correlation tests between soil physicochemical properties and the alpha-diversity of bacterial community measured in marsh soil with different yak excreta. The legend below is the color interval of different R values, and R < 0 indicates a negative correlation, while R > 0 indicates a positive correlation. * 0.01 < *P* ≤ 0.05, ** 0.001 < *P* ≤ 0.01, *** *P* < 0.001.

The Pearson correlation analysis revealed a significant positive link between moisture levels and the Ace index (0.001 < *P* ≤ 0.01), Shannon index (0.01 < *P* ≤ 0.05), and Chao1 index (0.001 < *P* ≤ 0.01) (Fig. 6). There was a notable positive association between the Simpson index (0.001 < *P* ≤ 0.01) and NO_3_^−^–N. Similarly, the Ace index (0.001 < *P* ≤ 0.01), Shannon index (0.001 < *P* ≤ 0.01), and Chao1 index (0.01 < *P* ≤ 0.05) showed a positive correlation with AP. The Ace index (*P* ≤ 0.001), Shannon index (0.001 < *P* ≤ 0.01), and Chao1 index (0.001 < *P* ≤ 0.01) showed a positive correlation with TOC. The Ace index (0.01 < *P* ≤ 0.05) and Chao1 index (0.01 < *P* ≤ 0.05) showed a positive correlation with TN.

In addition, the Simpson index was negatively correlated with moisture (0.01 < *P* ≤ 0.05), AP (0.001 < *P* ≤ 0.01), and TOC (0.001 < *P* ≤ 0.01). Ace index (0.01 < *P* ≤ 0.05), Shannon index (0.001 < *P* ≤ 0.01), and Chao1 index (0.01 < *P* ≤ 0.05) were negatively correlated with NO_3_^−^–N.

### Relationship between soil properties and bacterial community structure

**Fig 7:**
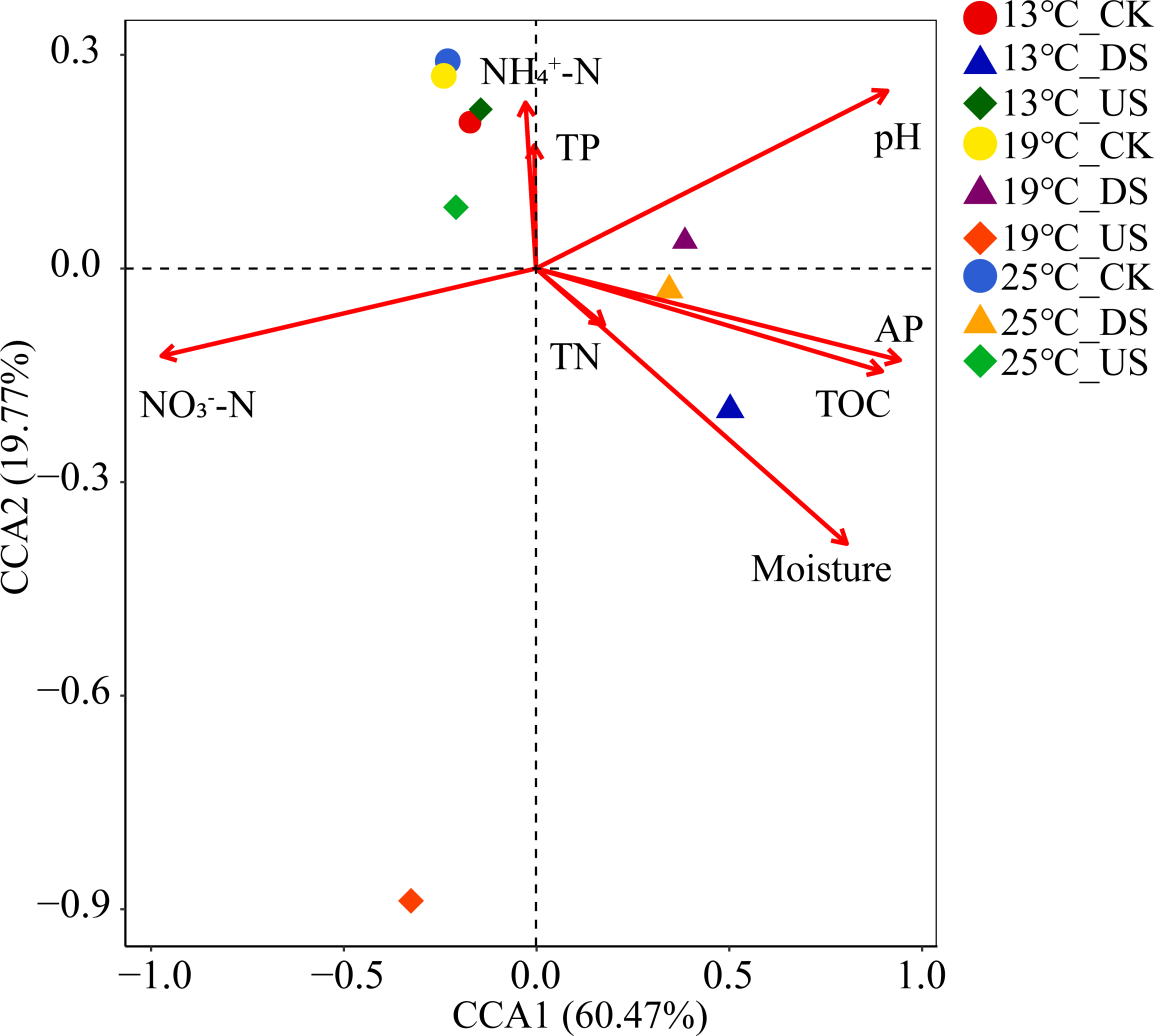
CCA between bacterial community structure and physical-chemical properties of marsh soil with yak excreta addition (CK, marsh soil; DS, yak dung + natural marsh soil; US, yak urine + natural marsh soil) under warming condition.

CCA dimensions explained 60.47% and 19.77% of the overall variance in bacterial community, respectively. Findings revealed that bacterial communities were mainly separated according to the different yak excreta treatment (Fig. 7). The primary influences on the bacterial community were NO_3_^−^–N, AP, pH, TOC, and moisture levels (*P* < 0.001) (see Table S4 at https://doi.org/10.5281/zenodo.17188844). The US treatments were separated at differing temperatures, predominantly affected by NO_3_^−^–N, while DS were mainly influenced by AP, pH, TOC, and moisture (*P* < 0.001).

**Fig 8:**
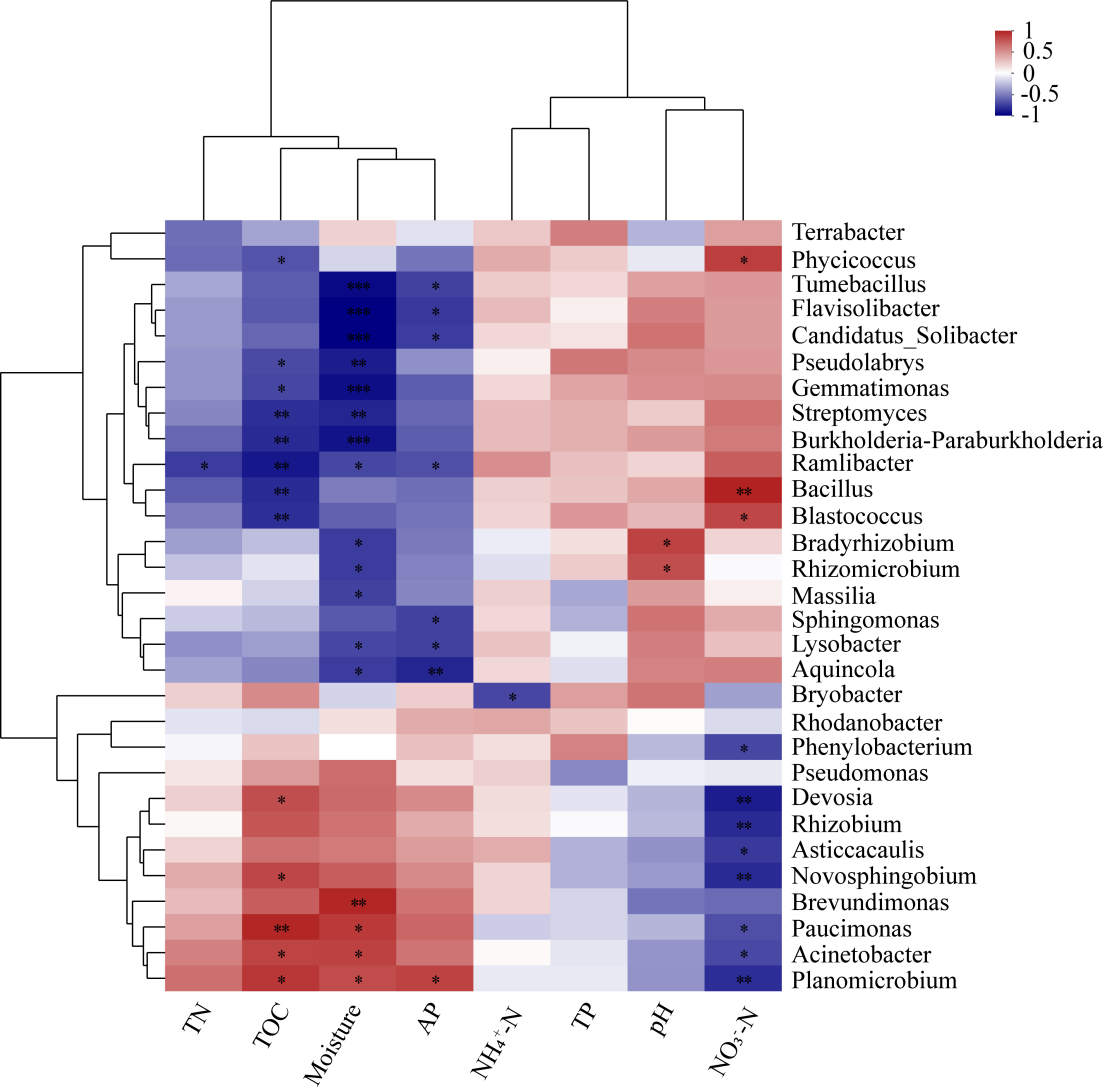
Heatmap of the correlation between dominant (the mean relative abundance of the top 30) bacterial genus and physical-chemical properties of marsh soil with yak excreta addition. The “R” value of the Spearman rank correlation coefficient is between −1 and 1 and is shown in different colors in the picture. The legend on the right is the color interval of different R values, and R < 0 indicates a negative correlation, while R > 0 indicates a positive correlation. * 0.01 < *P* ≤ 0.05, ** 0.001 < *P* ≤ 0.01, *** *P* < 0.001.

The correlation heatmap graphically illustrated the link between various bacterial genera and soil characters through the Spearman rank correlation coefficient (Fig. 8). It was observed that soil moisture, TOC, NO_3_^−^–N, and AP showed a negative correlation with 13, 8, 8, and 7 bacterial genera. TN showed a negative correlation with the relative abundance of *Ramlibacter* (0.01 < *P* ≤ 0.05). NH_4_^+^–N showed a negative correlation with the relative abundance of *Bryobacter* (0.01 < *P* ≤ 0.05). In addition, TOC, moisture, NO_3_^−^–N, pH, and AP showed a positive correlation with 5, 4, 3, 2, and 1 bacterial genera, respectively.

## DISCUSSION

### Yak excreta and temperature effect on soil properties

We found that both yak dung and urine significantly impact the marsh soil properties (Table 1). DS significantly increased the moisture, pH, TOC, and AP and decreased the NO_3_^−^–N, while US obviously increased the moisture, NH_4_^+^–N, and NO_3_^−^–N. Multiple researches support these findings, which are attributable to both direct and indirect impacts of adding yak excreta (9, 18, 25, 26). Firstly, yak excreta is rich in water, available carbon, nitrogen, and phosphorus, and these elements can be directly released into soils, enhancing both the nutrient availability and moisture of soils (25, 26). Secondly, the high concentration of organic substances and microbes in yak dung complicates the nutrient transfer and conversion process more than in yak urine following excreta addition in soil (7, 27). Moreover, yak dung could enhance other nutrients' availability and promote microbial activity in the soil (19, 20). Thirdly, the US markedly raised the levels of NO_3_^−^–N, aligning with findings from other studies (28, 29). This might be because of the rapid hydrolysis of urea in conjunction with the soil, followed by its oxidation into NO_3_^−^–N throughout the nitrification phase (9). In contrast, DS obviously decreased NO_3_^−^–N concentrations. It might be indicated that nitrification was inhibited by dung application (9). The findings revealed that the impact of urine addition on altering soil environmental conditions differed slightly from the effects of dung addition. Fourthly, both yak dung and urine contain higher pH values compared to the marsh soil. Yak dung directly raised the soil pH value, while yak urine had no significant effect on the soil pH value (Table 1). It is further indicated that the impacts of yak dung and urine on the characteristics of marsh soil are different.

In the current study, the pH of CK, DS, and US was significantly higher at 13°C than at 19°C (*P* < 0.05). In contrast, NH_4_^+^–N of CK was higher at 25°C than at 13°C, while that of US was higher at 13°C than at 25°C (*P* < 0.05) (Table 1). These results indicated that the response of pH to temperature does not change with or without the yak excreta addition, while the trend of NH_4_^+^–N is the opposite. It is further indicated that there are some interactions of temperature and yak excreta impacted on soil properties. Yak excreta and temperature interactions significantly impacted pH and NH_4_^+^–N (*P* < 0.01) of marsh soil, further supporting this observation (see Table S2 at https://doi.org/10.5281/zenodo.17188844).

### Yak excreta and warming effect on soil bacteria

#### Yak excreta and warming effect on soil bacterial diversity

In the current study, the unique OTUs of US and DS at three different temperatures were lower than those of CK. These findings suggested that yak excreta addition could result in a reduction of distinct OTUs. The introduction of yak excreta could have altered the microenvironment of marsh soil, subsequently diminishing species’ distinct OTUs. In this study, Ace and Chao1 indices of DS were lower than those of CK, indicating that yak dung decreased the bacterial richness of marsh soil. The Shannon index of DS was lower than CK, and the Simpson index was higher than CK, indicating that yak dung decreased the bacterial diversity of marsh soil. However, previous studies have shown that dung deposition could increase microbial richness and diversity through the introduction of numerous novel microbial species and the augmentation of nutrient availability (1, 27). The observed reduction in abundance and variety of bacterial populations might be ascribed to the diminution of rare species according to the influx of nitrogen, culminating in a decreased biodiversity of microorganisms overall (30). In addition, dung deposition led to the demise of numerous microbial species due to escalating antibiotic pollution (31, 32). In contrast, the US had minimal impact on the richness and variety of bacterial community. The only exception was that the Ace and Chao1 indices of the US were lower than those of CK at 19°C. It shows that the response of the soil bacterial community to yak dung is more sensitive than yak urine.

In the present study, the Ace, Chao1, and Shannon indices of CK at 25°C exceeded those at 13°C, while the Simpson index of CK at 25°C fell below those at 13°C, suggesting greater bacterial richness and diversity in marsh soil at 25°C compared to 13°C. The Shannon index of DS at 13°C was lower than at 19°C and 25°C, but the Simpson index had an opposite trend, indicating that the bacterial diversity of DS at 13°C was lower than at 19°C and 25°C. It further shows that higher temperature relatively increases the richness and variety of bacterial populations in marsh soil.

#### Yak excreta and warming effect on soil bacterial community structure and co-occurrence network

Findings of this research indicate that *Proteobacteria*, *Actinobacteriota,* and *Chloroflexi* predominated as phyla in marsh soils under varied treatments, aligning with primary patterns of dominant bacteria in soil ecosystems (2, 18). Our research indicated DS led to a rise in the proportion of *Proteobacteria* and *Firmicutes*, but a decrease in the proportion of *Chloroflexi*, *Acidobacteria*, and *Gemmatimonadetes*. In contrast, US led to a rise in the proportion of *Firmicutes* and a decrease in the proportion of *Proteobacteria* and *Chloroflexi* (Fig. 3). These changes might be caused in multiple ways. Firstly, the incorporation of yak excreta represents a common process of community merging, capable of altering the microbial community structure through the taxon addition phenomenon (18, 33). Wang et al. discovered that *Proteobacteria* and *Firmicutes* were the predominant bacterial phyla in dung (34). DS enhanced the relative abundance of *Proteobacteria* and *Firmicutes*, indicating that the effect of taxon addition was somewhat influential in modifying the soil microbial community due to yak dung addition. Secondly, yak excreta addition markedly enhanced the nutrient levels in soil, thereby benefiting microbial copiotrophic groups (35, 36). According to our study, DS significantly increased the relative abundance of various copiotrophic species, including *Proteobacteria*, *Firmicutes,* and *Bacteroidota*. Conversely, DS decreased the population of various oligotrophic microbial groups, including *Chloroflexi* and *Acidobacteriota*.

The bacterial communities were mainly separated according to the different yak excreta treatments (Fig. 5). It is indicated that yak excreta addition is the main cause of changing the bacterial community. In addition, the samples of US and DS at 13°C were, respectively, separated at 19°C and 25°C (Fig. 5), indicating that warming significantly regulated the bacterial community in marsh soil with yak excreta addition. Warming impacts soil microbial communities by changing soil temperature and moisture and regulating soil carbon consumption and the absorption patterns of carbon by microorganisms (37, 38). Notably, the relative abundance of *Acinetobacter* in DS at 13°C was higher than that at 19°C and 25°C. The proportion of *Sphingomonas* in US showed a decreasing trend: 13°C > 19°C > 25°C. It further suggests that the bacterial community of marsh soil with yak excreta addition is sensitive to climate warming.

Yak excreta significantly decreased the relative abundance of *Candidatus solibacter, Flavisolibacter*, *Gemmatimonas*, and *Massilia* (see Fig. S1 at https://doi.org/10.5281/zenodo.17188844). *Candidatus solibacter* possesses the ability to break down organic substances and utilize carbon sources. It associates with the organic matter positively and impacts the conversion of nitrogen in soil directly (2, 39). *Flavisolibacter* was regarded as advantageous, significantly contributing to the enhancement of plant disease resistance and fostering plant development and carbon dioxide fixation (40). Plant growth can be enhanced by *Gemmatimonas* (41). *Massilia* has many functions, such as participating in soil carbon and nitrogen cycling, sewage denitrification, degrading polycyclic aromatic hydrocarbons, and enhancing plant stress resistance (42). Remarkably, the common characteristics of these bacterial genera are playing a positive effect in maintaining soil health, promoting plant growth, and degrading pollutants. Meanwhile, it also indicates that yak excreta not only significantly changes the soil bacterial community structure but also damages the normal function of marsh soil.

In the current study, the bacterial co-occurrence network for DS had more nodes and edges and shorter average path lengths than those for US and CK, indicating greater complexity. The low average path length in the DS network indicates faster information transmission within the network, allowing the bacterial community to respond to environmental change more quickly (43). Liu et al. discovered that dung deposition reduced the microbial co-occurrence network complexity in alpine grassland soil (18). However, our research showed that dung addition increased the bacterial co-occurrence network complexity of the bacterial community in alpine marsh soil. It indicates that microbial interactions might be significantly various in different soil environments. We observed a reduction in the complexity of the bacterial co-occurrence network due to urine addition. The less complex bacterial co-occurrence network could be partially caused by the increased nitrogen availability with urine addition (44, 45).

The co-occurrence network reveals the species coexisting in the niche and identifies the microbial taxa that have the highest effect on the microbial community (46). Yak excreta obviously altered interactions among microorganisms, and there were differences in the variations in interactions in DS and US (Fig. 5 and Table 2). Both DS and US showed fewer negative interactions and more positive interactions than CK. It is suggested that yak excreta has dual effects on the interactions between synergistic and antagonistic taxa (47).

### Correlation between properties and bacterial community in marsh soil with yak excreta addition under warming conditions

The current research revealed a clear link between the bacterial population and the physicochemical characteristics of marsh soil when yak excreta were added under warming conditions, verifying that alterations in the characteristics of marsh soil due to yak excreta under warming conditions influence the bacterial community composition and structure (Fig. 6). Environmental elements showed either an inverse or direct correlation with the prevalence of various bacteria (Fig. 8). It further indicates that alterations in environmental conditions can have either detrimental or beneficial impacts on specific species, influencing the entire structures of soil bacterial communities (11, 48, 49).

According to our study, NO_3_^−^–N, AP, pH, TOC, and moisture are major factors affecting bacterial communities (Fig. 6, also see Table S4 at https://doi.org/10.5281/zenodo.17188844). Previous studies have also revealed that pH, AP, TOC, soil water capacity, and available N are vital elements that significantly influence the soil bacterial community structure (16, 50). Our findings revealed that NO_3_^−^–N was the dominant factor influencing soil bacterial communities in the US. NO_3_^−^–N showed a negative correlation with the Ace, Shannon, and Chao1 index, and a positive correlation with the Simpson index. Moreover, NO_3_^−^–N positively affected three bacterial genera but negatively affected eight bacterial genera (Fig. 8). It is widely believed that pH is a key factor influencing microbial communities in soil (18, 51). Therefore, increasing the pH of yak dung and decreasing the pH with warming could effectively change the composition and structure of microbial communities. This is confirmed by the significant relationships between soil pH and the relative abundance of *Bradyrhizobium* and *Rhizomicrobium* (Fig. 8). Moreover, *Acidobacteria* is acidophilic bacteria and sensitive to soil pH change. In current research, DS decreased the proportion of *Acidobacteria* significantly; it might be because of the increase in soil pH with yak dung addition. The moisture is also important in determining microbial community changes (52). In this study, the moisture increased by yak excreta addition and decreased with warming, which could change the microbial community. There is a significant relationship between moisture and microbial community (Fig. 7). Moreover, moisture positively affected four bacterial genera but negatively affected 13 bacterial genera (Fig. 8). TOC is considered a key factor affecting soil microbial communities (9, 53). Therefore, DS increased TOC could significantly alter the composition and structure of bacterial community. It is further confirmed by the increment in the proportion of *Firmicutes. Firmicutes* are associated with organic substance degradation (54), and their proportion increases with TOC. There was an obvious correlation between AP and microbial community (Fig. 7). AP showed a positive correlation with Ace, Shannon, and Chao1 index, and a negative correlation with Simpson index (Fig. 6). Moreover, AP positively affected one bacterial genus but negatively affected seven bacterial genera (Fig. 8).

### Conclusion

This study showed that yak excreta have a stronger impact on soil characteristics, bacterial community diversity, and composition than temperature. Warming increased the alpha-diversity of bacterial community in marsh soil, yak dung had an opposite effect, while yak urine exerted almost a negligible effect. Yak excreta exhibited a significant impact on the bacterial community structure in marsh soil, and warming obviously regulated these effects. Yak dung altered more bacterial genera of marsh soil than yak urine. Yak dung increased the complexity of the bacterial co-occurrence network. Moreover, yak dung obviously strengthened the bacterial association interaction in marsh soil, while yak urine had the opposite trend. Yak excreta and temperature regulated the bacterial communities by altering NO_3_^−^–N, AP, pH, TOC, and moisture of marsh soil. Our findings could provide novel insights into the different effects of yak dung and urine on bacterial communities in marsh soil and highlight that the impacts of yak excreta on bacterial communities are sensitive to climate warming. Findings and suggestions derived from the current study provide critical information for guiding livestock excreta management in the background of climate warming, thereby promoting a healthy and sustainable development of the alpine marsh ecosystem.

## Data Availability

Sequencing data have been deposited in the NCBI database under accession number SRP627120. Supplementary data for this article can be found online at https://doi.org/10.5281/zenodo.17188844.
